# Using Adult Learning Theory to Guide Instruction in Training Physical Therapy Students on the Use of AI in Didactic Education

**DOI:** 10.1007/s40670-024-02066-0

**Published:** 2024-05-18

**Authors:** Stacia Hall Thompson, Patricia Fecher

**Affiliations:** 1 Physical Therapy Department, Methodist University, Fayetteville, NC USA; 2 Education Department, Methodist University, Fayetteville, NC USA

**Keywords:** Artificial intelligence, Adult Learning Theory, Physical therapy education, Critical thinking

## Abstract

Artificial intelligence (AI) use is increasing in physical therapy. Didactic curricular experiences can introduce AI to prepare students for clinical practice. A targeted approach to learning incorporating Adult Learning Theory can foster student development of critical thinking for translation to appropriate use in clinical practice.

Artificial intelligence (AI) is a trending subject in physical therapy. With the technology boom and the emergence of AI applications aiding clinical practice, physical therapists (PTs) can use AI to predict service needs and outcomes, analyze data, track patient performance, develop diagnoses, and document/bill for services rendered [1, 2].

PTs must understand how AI works and the considerations needed when analyzing AI-system outputs. Results evaluation is essential for the correct application to patient care, noting the necessity of PTs when incorporating AI into practice [1]. Without necessary information analysis, the potential for improper application of information exists [1, 2].

In a recent survey, nearly 62% of PTs indicated they did not know about AI, and approximately 72% indicated that AI should be taught in the classroom [2]. To combat the improper application of information, lack of awareness, and rapid growth/use of AI, PTs must learn to use AI appropriately. Initiating opportunities to incorporate critical thinking in didactic curricula would equip a future workforce with the necessary skills to analyze AI output and optimize findings to enhance patient outcomes [1, 2]. This innovative article discusses intentionally incorporating an orientation activity for first-year physical therapy students (SPTs) to learn the necessity of critical thinking when using AI. Through this one learning activity example surrounding AI use, SPTs can apply learned skills for future professional practice.

Including AI within instruction requires modifying traditional pedagogical methods. Thus, the authors used the Adult Learning Theory (ALT) Teaming Pillar Practice (TPP) to support the growth and learning of adults with diverse ways of thinking. ALT allows individuals to use experience, roles/responsibilities, and motivations to foster understanding. TPP uses open communication, decreases isolation, builds interdependent relationships, and supports adult development through balanced conversation through a collaborative approach to synthesize the generated AI output for the correct application to patient care [2, 3].

Figure 1 illustrates the activity process. First, initial instruction focused on AI generators and technology use. Next, SPT teams evaluated AI-generated responses through guiding questions to support their shifting and growth in understanding the importance of critical thinking. Students then entered a PT-specific protocol into the AI generator. Using Teaming, students explored their thinking, perspectives, and assumptions, evaluating the “thinking” produced by their AI query [3]. Lastly, SPTs reflected on the outcomes of this learning activity for application to future practice.

**Fig. 1:**
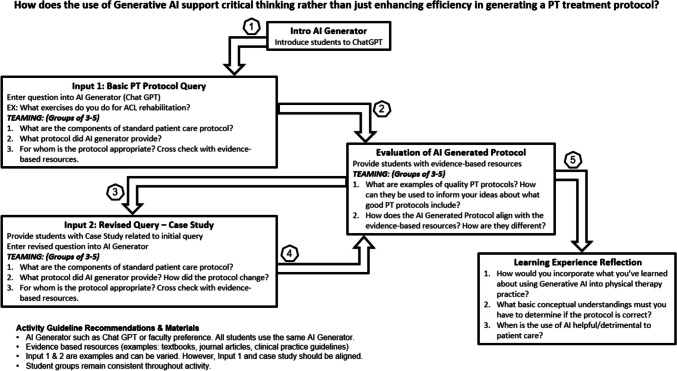
Orientation activity stages and TPP questions

The orientation activity highlighted the importance of critical thinking when using AI. Follow-up occurred later in the semester, and two-thirds of the SPTs taught responded that they had used AI, with approximately 50% indicating that they now incorporate fact-checking. The following trends emerged from the learning experience: (1) Asking intentional/specific clinical questions is essential, (2) the importance of asking questions with patient-specific details, (3) AI efficiently synthesizes information; however, SPTs need to spend time to ensure the protocol is correct, and (4) fact-checking is necessary to ensure the AI-generated response was correct and could be applied to studies or clinical care. Through this orientation activity, SPTs learned that most questions in the clinical field are ambiguous and that every response depends on collecting evidence and patient information. Lastly, when reflecting on their learning, STPs responded that it is good to teach AI because it is becoming more prominent, and it was insightful to see how AI is applied in the clinical setting.

This activity introduced STPs to one-way AI aids PTs in clinical practice. Even though half of the students instructed now incorporate fact-checking, the goal of all users engaging in critical thinking still needs to be met. Students may not use AI to fact-check 100% of the time because traditional didactic assignments do not encourage the utilization of AI. Experiences in isolation do not transfer into consistent application and practice. Therefore, SPTs need ongoing, embedded opportunities aligned to all levels of didactic experiences for full incorporation into daily practice. Thus, the curriculum must change to fully equip students with the skills necessary to practice in today’s workforce.

This activity provides a structured process for implementation throughout didactic training to prepare SPTs for clinical practice. Several areas arise to improve future training on AI use. Expanded implementation of learning activities incorporating AI throughout the curriculum would benefit the development of critical thinking. In addition, further analysis of the experience design with a partner or independent work may yield different results. Lastly, faculty may incorporate reflection on the output of different AI generators to see if similar results are attained.
